# Impact of Dry Eye Disease and Lipid-Containing Artificial Tears on Keratometric Reproducibility and Intraocular Lens Calculation in Cataract Patients

**DOI:** 10.3390/medicina62010179

**Published:** 2026-01-15

**Authors:** Valentina Lacmanović Lončar, Danijel Mikulić, Vedrana Aljinović-Vučić, Zoran Vatavuk, Ivanka Petric Vicković

**Affiliations:** 1 Department of Ophthalmology, Sestre Milosrdnice University Hospital Center, 10000 Zagreb, Croatia; mikulicdanijel@gmail.com (D.M.);; 2 Medical Affairs Department, Jadran Galenski Laboratorij d.d., 51000 Rijeka, Croatia; 3 Department of Basic and Clinical Pharmacology and Toxicology, School of Medicine, University of Rijeka, 51000 Rijeka, Croatia; 4 School of Dental Medicine, University of Zagreb, 10000 Zagreb, Croatia

**Keywords:** dry eye disease, cataract surgery, keratometry, intraocular lens

## Abstract

*Background and Objectives*: Tear film instability and corneal surface irregularity are important sources of variability in keratometric and corneal topographic measurements, particularly affecting astigmatic magnitude and axis. Accurate preoperative biometry is crucial for optimal refractive outcomes in cataract surgery. Dry eye disease (DED) may compromise the reproducibility of keratometric parameters, leading to errors in intraocular lens (IOL) power calculation. This study aimed to evaluate the impact of DED on the reproducibility of keratometric measurements and to assess the effect of a four-week treatment with lipid-containing artificial tears on these parameters in cataract patients. *Materials and Methods*: This cross-sectional study included 116 patients scheduled for cataract surgery, of whom 65 (56.0%) had DED and 51 (44.0%) served as controls. All patients underwent two preoperative keratometric measurements 10–20 min apart (IOL1 and IOL2). The control group proceeded to surgery the next day, while surgery in the DED group was postponed. Patients with DED received preoperative therapy with lipid-containing artificial tears. Follow-up assessments occurred one month after therapy (keratometric measurement named IOL3) and eight weeks postoperatively. Clinical evaluation included slit-lamp examination, dry eye testing according to Dry eye Workshop II (DEWS II) criteria: Ocular surface Disease Index (OSDI), Tear Break-Up Time (TBUT), Schirmer I, Oxford staining, and meibomian gland assessment), ocular biometry, and postoperative spherical equivalent measurement using an auto ref-keratometer. Nonparametric statistical analyses were applied to evaluate associations between parameters. *Results*: In the DED group, corneal astigmatism showed a significant difference between IOL1 and IOL2 (Wilcoxon signed-rank test {Z = 2.43; *p* = 0.015}). Significant changes in predicted IOL power were observed between pretreatment and posttreatment values (t = 2.57; *p* = 0.013) and between IOL2 and IOL3 (t = 2.23; *p* = 0.029), indicating improved keratometric stability following tear film therapy. No additional significant correlations were identified. *Conclusions*: DED adversely affects the reproducibility of keratometric measurements and may compromise IOL power selection. Preoperative identification and treatment of DED, followed by repeated biometry after tear film stabilization, are strongly recommended to enhance refractive accuracy and optimize surgical outcomes in cataract patients.

## 1. Introduction

Cataract surgery is among the most frequently performed surgical procedures worldwide, with patient expectations for excellent postoperative refractive outcomes continuously rising. Despite the evolution of biometry devices and modern intraocular lens (IOL) power calculation formulas, refractive errors greater than ±0.5 diopters (D) are still reported in approximately 20% to 40% of all cases [1,2,3,4,5,6].

Since keratometry constitutes a fundamental component of biometry, any error in the assessment of corneal curvature directly translates into inaccurate IOL power calculation and refractive surprise after surgery [6]. In addition, keratometry-related measurement errors have been reported to account for approximately 22% of inaccuracies in IOL power calculation, underscoring the need for highly reproducible corneal measurements [7].

Keratometric measurements rely on the integrity of the tear film, as optical biometers such as the IOL Master assess reflection from the air–tear film interface, rather than from the corneal epithelium itself [8]. Therefore, a stable tear film is essential to achieve reproducible readings. Even minor fluctuations in tear film thickness or regularity can cause variability in corneal curvature, ultimately leading to postoperative refractive inaccuracy.

Dry eye disease (DED) is a multifactorial condition characterized by tear film instability, hyperosmolarity, and ocular surface inflammation, resulting in discomfort and visual fluctuation. Reported prevalence ranges widely, from 5% to 50%, and increases with age [9], making DED particularly relevant in the cataract population. Tear film instability and surface irregularity are known to induce significant measurement noise in keratometry and corneal topography, particularly affecting astigmatic axis and power [10,11,12].

Elevated tear osmolarity has been shown to increase variability in keratometric measurements, resulting in less predictable IOL power calculations [10]. Tear film disruption similarly contributes to a rise in higher-order aberrations and a decline in optical quality [13]. These findings underline the importance of optimizing the ocular surface before performing biometric measurements.

Artificial tears are commonly applied to restore tear film regularity prior to biometry; however, their immediate influence on corneal measurements remains controversial. Studies have demonstrated that both low- and high-viscosity artificial tears can significantly increase short-term variability in keratometric readings, with effects lasting up to five minutes after instillation [6]. Moreover, measurable alterations in ocular aberrations may persist for up to ten minutes following the application of sodium hyaluronate [10]. These transient effects highlight the need to determine the optimal timing of biometry after the use of lubricants.

Evidence highlights the importance of targeting the lipid layer in evaporative forms of dry eye associated with Meibomian gland dysfunction (MGD) [13], while different artificial tear formulations have been shown to variably influence tear film stability and optical quality [14]. Evidence highlights the importance of targeting the lipid layer in evaporative forms of dry eye associated with MGD [3], and lipid-containing artificial tears have been shown to restore tear film stability and improve tear breakup time (TBUT) in patients with evaporative dry eye [15,16]. While clinical trials have shown improvement in subjective symptoms and objective surface parameters, their effect on the stability of keratometric values and the accuracy of IOL power prediction remains underexplored.

Given the growing demand for refractive precision in cataract surgery, understanding the interplay between DED, artificial tear therapy, and keratometric stability is crucial. The present study therefore aims to evaluate the influence of DED on the reproducibility of keratometric parameters used for IOL power calculation, and the effect of a four-week treatment with lipid-containing artificial tears on these parameters in cataract patients with DED. By clarifying whether targeted tear film therapy can enhance measurement reliability and reduce refractive prediction error, this research seeks to refine preoperative assessment protocols and improve refractive outcomes following cataract surgery.

## 2. Materials and Methods

This cross-sectional study was carried out at the Sestre milosrdnice University Hospital Centre. The study participants were patients scheduled for cataract surgery, and the period of data collection was from December 2024 to July 2025. All study participants had to read the informed consent document and certify that they had read, understood, and agreed to participate in the study. The Ethics Committee of the Sestre milosrdnice University Hospital Centre Zagreb approved the study in accordance with the principles of the Declaration of Helsinki (protocol code 003-06/25-03/007 number 251-29-11/3-25-28; date of approval: 11 February 2025).

All patients underwent a complete ophthalmologic examination the day before cataract surgery. The ophthalmologic examination included slit-lamp examination, dry eye examinations according to the Dry eye Workshop II (DEWS II) criteria: Ocular surface Disease Index (OSDI), Tear Break-Up Time (TBUT), Schirmer I, positive staining based on the Oxford scheme, tests for Meibomian gland function and ocular biometry Based on the examination, patients were divided into two groups: patients with dry eye disease (DED group) and the control group. Patients were identified as belonging to the DED group if, in addition to positive symptoms, they had at least one out of four objective dry eye measurements.

Inclusion criteria were age above 18 years and indication for age-related cataract surgery. Patients were excluded if they met any of the following criteria: history of herpes keratitis or varicella-zoster keratitis; Sjögren’s syndrome; history of Stevens–Johnson syndrome; use of systemic or topical steroid therapy; current dry eye treatment (artificial tears, cyclosporine, corticosteroids); active infection; patients who had undergone keratorefractive procedures or other ocular surgeries in the past 3 months; patients with corneal scarring; or patients with other ocular surface pathologies. Patients in whom IOL power could not be calculated using the IOLMaster 700 (Carl Zeiss Meditec AG, Jena, Germany) due to severe cataract or poor cooperation were excluded. A priori sample size calculation was performed to ensure a 95% confidence level, indicating that a minimum of 95 participants was required for the study.

Patients with DED followed a three-visit protocol. The first examination for all patients (both the DED group and the control group) was performed the day before cataract surgery. Optical biometry was first performed in all patients before the use of anesthetic and mydriatic drops in order to avoid any possible influence on corneal parameters. All measurements were performed by a single examiner using the IOLMaster 700. Two measurements were taken 10 to 20 min apart (IOL1 and IOL2), and flat and steep K (K1 and K2), average keratometry (Avg K), corneal astigmatism (Ast K), and the absolute differences between the first and second measurements were analyzed. The IOL power was calculated using the Barrett Universal II formula. Patients for whom implantation of a toric IOL was indicated were excluded from the study. Target refraction for IOL power calculation was emmetropia.

The assessment of subjective DED symptoms was performed using the Ocular Surface Disease Index (OSDI). The OSDI questionnaire was completed between the first and second optical biometry measurements, while the dry eye examinations were conducted after the second optical biometry. TBUT was evaluated using fluorescein strips (Optitech Eyecare: Fluorescein Sodium Ophthalmic Strips USP, Prayagraj, India), which were moistened with preservative-free saline and placed in the lower fornix. A TBUT value of less than 7 s was considered positive. Ocular surface staining was graded according to the Oxford scale (0–5). For the Schirmer I test, the strip was placed in the lower fornix at the junction of the lateral and middle third of the lower eyelid. After 5 min, the strips were removed; values below 10 mm were indicative of dry eye.

Meibomian gland function was assessed by evaluating Meibomian gland expressibility (0–3) and the quality of expressed meibum (0–3). MGD was diagnosed when expressibility was >1 and meibum quality was >1.

The control group underwent cataract surgery the following day, while cataract surgery in the DED group was postponed. In the control group, the first biometric data from the IOL Master measurement were used to select the IOL that was implanted (IOL1). All patients in the DED group received preoperative therapy with artificial tears—lipid-containing artificial tears Vizol S Hydro Lipid Balance (Vizol S HLB), Jadran Galenski Laboratorij d.d. (JGL), Rijeka, Croatia—four times per day for one month. Vizol S Hydro Lipid Balance is a lipid-containing artificial tear formulation composed of 0.25% castor oil, 0.15% hyaluronic acid, 1% glycerol, and 0.5% vitamin E TPGS 1000, designed to support the lipid, aqueous, and mucin layers of the tear film. The second visit for the DED group took place one month after completing the therapy, optical biometry was repeated using the same device, and the IOL power was calculated using the Barrett Universal II formula on the day of the surgical procedure. In the DED group, the biometric data from the IOL Master at the second visit (after the DED therapy) were used to select theIOL (IOL3). In patients with DED, dry eye parameters including Ocular Surface Disease Index (OSDI), tear breakup time (TBUT), Oxford grading, Schirmer I test, and Meibum Quality Score (MQS) were repeated after four weeks of treatment with lipid-containing artificial tears. All cataract surgeries were performed by two experienced surgeons (authors V.L.L. and I.P.V.) under topical anesthesia. If a patient underwent cataract surgery in both eyes, only the first operated eye was included in the analysis. In all patients, a one-piece aspheric monofocal IOL (Tecnis PCB00, Johnson & Johnson Surgical Vision, Santa Ana, CA, USA) was implanted. At the end of the surgical procedure, intracameral cefuroxime was administered in all patients. Postoperatively, all patients received topical corticosteroid therapy four times daily for three weeks, and patients in the DED group continued therapy with artificial tears four times daily for ten weeks. The third visit took place 8 weeks after the surgery, and it included visual acuity, subjective refraction, absolute prediction error, and repeated DED tests (OSDI, TBUT, Schirmer test, ocular surface staining, and analysis of Meibomian gland function. Postoperative spherical equivalent refraction was measured using an auto ref-keratometer (Topcon KR 8900, Topcon Haelthcare, Capelle aan den Ijssel, The Netherlands). Prediction error (PE) is calculated as the difference between the postoperative spherical equivalent and the predicted spherical equivalent obtained from the IOL calculation using the Barrett Universal II formula. Since the prediction error can have both positive and negative values, the absolute prediction error was used. The mean absolute error (MAE) was compared between the DED and the control group.

## 3. Results

### 3.1. Study Population and Dry Eye Classification

A total of 116 eyes from 116 patients were included, with 65 patients (56.0%) classified as having dry eye disease (DED) and 51 (44.0%) serving as controls. Baseline demographic characteristics, including age and sex distribution, are shown in Table 1. Within the DED group, the proportions of aqueous-deficient, evaporative, and mixed dry eye, together with the distribution of symptom severity, are summarized in Table 2.

### 3.2. Baseline Keratometry: DED Versus Controls

**Figure 1:**
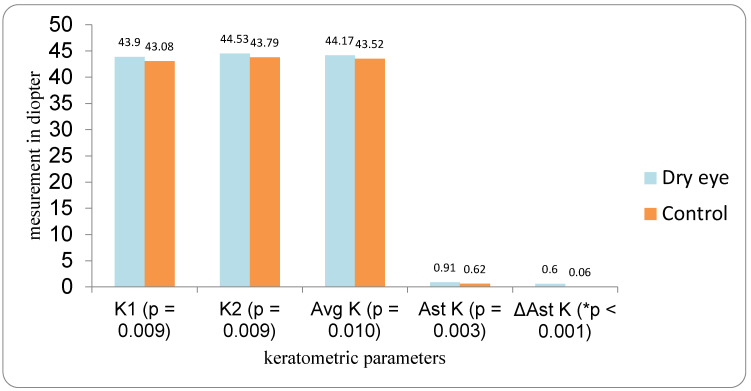
Comparison of parameters of DED vs. Control group. * statistically significant difference.

Baseline keratometric values differed significantly between groups (Figure 1). In the DED group, mean flat keratometry (K1) was 43.90 D, steep keratometry (K2) 44.53 D, and average keratometry (Avg K) 44.17 D, compared with 43.08 D, 43.79 D, and 43.52 D, respectively, in the control group. These differences were statistically significant (K1: *p* = 0.009; K2: *p* = 0.010; Avg K: *p* = 0.003). Mean corneal astigmatism (Ast K) was higher in the DED group (0.91 D) than in controls (0.62 D, *p* < 0.001). The change in astigmatism between measurements (ΔAst K) was also greater in the DED group (0.60 D) than in the control group (0.06 D, *p* = 0.009). 

**Figure 2:**
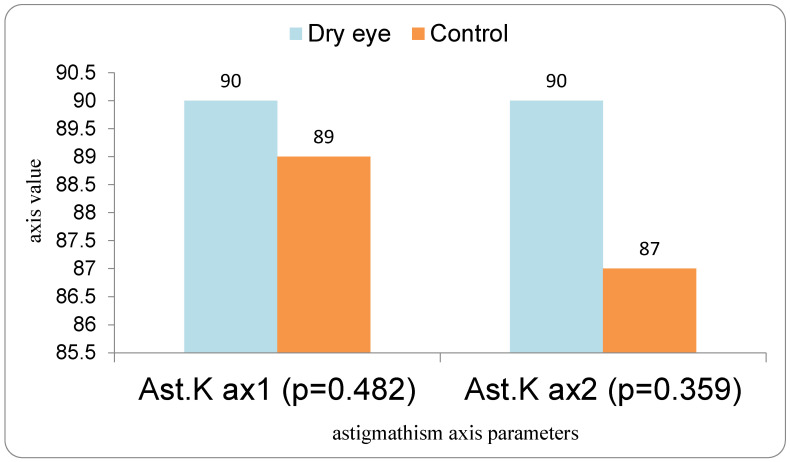
Mean astigmatism axis orientation in IOL1 and IOL2 in the DED and control groups.

In contrast, comparison of astigmatic axis (Ast K axis 1 and 2), as well as K1 and K2 axes at different measurements (Figure 2.), showed no statistically significant differences (all *p* > 0.05, Mann–Whitney U test).

**Figure 3:**
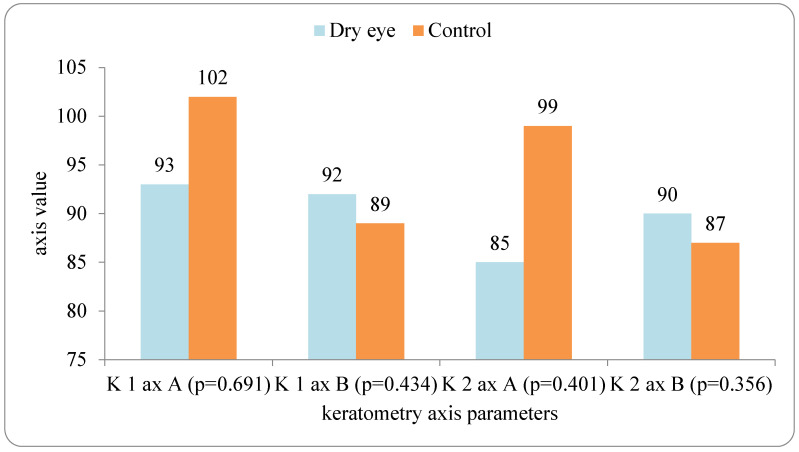
Axis comparison in two measurements (A and B).

Keratometric axes did not differ significantly between groups. (all *p* > 0.05, Mann–Whitney U test; Figure 3).

### 3.3. Repeatability of Keratometry in the DED Group

**Figure 4:**
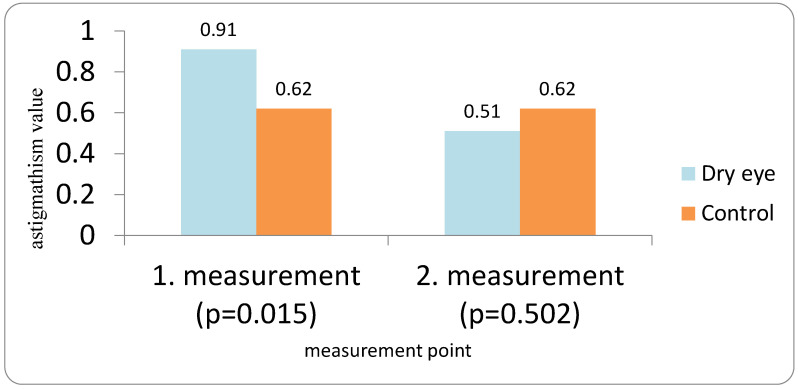
Corneal astigmatism change in DED vs. Control group in repeated preoperative measurements.

In the DED group, corneal astigmatism showed a significant change between the first and repeated preoperative measurements (Figure 4). Mean Ast K decreased from 0.91 D at the first measurement to 0.51 D at the second measurement (Wilcoxon signed-rank test {Z = 2.43; *p* = 0.015}). In the control group, mean Ast K remained stable at 0.62 D for both measurements (Wilcoxon signed-rank test {Z = 0.67; *p* = 0.502}).

**Figure 5:**
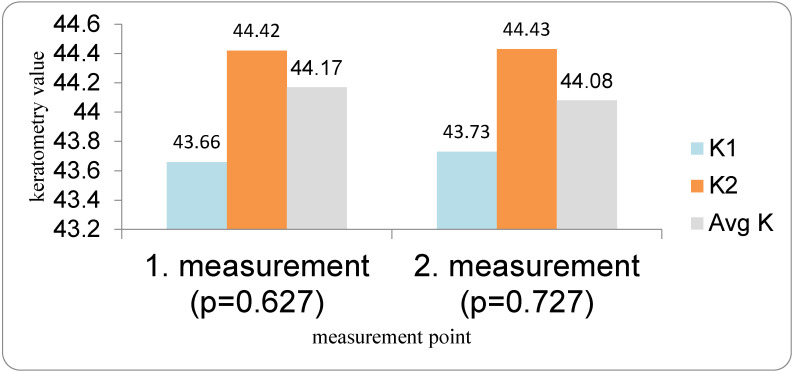
K-values in DED group in repeated preoperative measurements.

In contrast, spherical keratometric values in the DED group were relatively stable between measurements (Figure 5). Mean K1 changed from 43.66 D to 43.73 D, K2 from 44.42 D to 44.43 D, and Avg K from 44.17 D to 44.08 D between the first and second preoperative measurements. None of these differences reached statistical significance (K1: *p* = 0.067; K2: *p* = 0.727; Avg K: *p* = 0.126; Student’s paired *t*-test).

**Figure 6:**
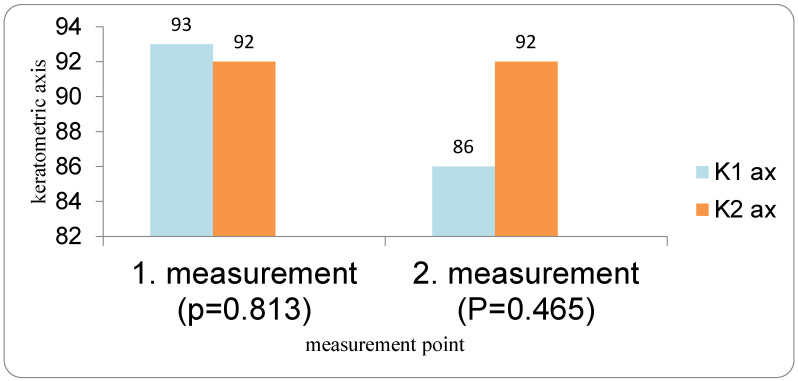
K-axis values in DED group in repeated preoperative measurements.

Keratometric axes in the DED group (K1 and K2 axis) also remained stable between the first and second measurements (*p* = 0.813 and *p* = 0.465, respectively; Wilcoxon test). Similarly, in the control group, there were no significant changes in astigmatic or keratometric axes between repeated measurements (all *p* > 0.05, Wilcoxon test). Although mean spherical keratometric values (K1, K2, Avg K) remained stable, corneal astigmatism, which is derived from the difference between K2 and K1 and influenced by axis orientation, showed greater variability, explaining the apparent discrepancy between stable mean keratometry and changing astigmatism (Figure 6).

### 3.4. IOL Power Calculation

In the DED group, predicted IOL power differed slightly across the preoperative keratometric measurements. Mean calculated IOL power increased from 21.99 ± 1.99 D at the first measurement to 22.00 ± 1.98 D at the second measurement, and to 22.11 ± 1.96 D at the follow-up assessment after treatment. In the control group, predicted IOL power remained stable between the two preoperative measurements (21.61 ± 3.09 D and 21.66 ± 2.98 D). The distribution of implanted Tecnis PCB00 IOLs (range 18–24 D) did not differ significantly between groups (*p* > 0.05).

Within the DED group, statistically significant differences were observed between IOL 1 and IOL 3 (t = 2.57, *p* = 0.013) and between IOL 2 and IOL 3 (t = 2.23, *p* = 0.029), indicating a modest shift in predicted IOL power following tear film treatment.

### 3.5. Postoperative Refractive Outcomes and Prediction Error

**Figure 7:**
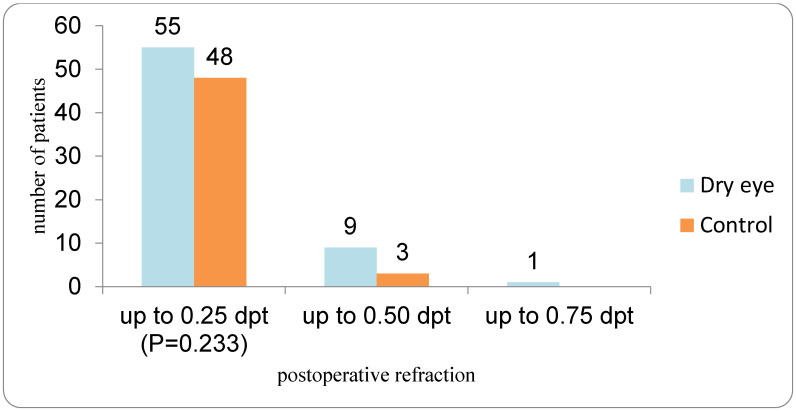
Postoperative refractive outcomes in DED vs. Control group.

Postoperative spherical equivalent refraction showed similar accuracy between the DED and control groups (Figure 7). In the DED group, 55 eyes achieved a postoperative refraction within ±0.25 D, 9 eyes within ±0.50 D, and 1 eye within ±0.75 D of the intended target. In the control group, 48 eyes were within ±0.25 D and 3 eyes within ±0.50 D; no eyes had a residual error exceeding ±0.50 D up to the ±0.75 D category. The distribution of postoperative refraction did not differ significantly between groups (χ^2^ = 1.42; *p* = 0.233).

**Figure 8:**
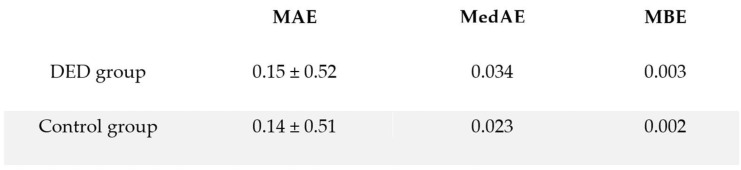
Absolute prediction error in DED vs. Control group.

Absolute prediction error metrics after tear film treatment in the DED group were comparable to those of the control group (Figure 8). Mean absolute error (MAE) was 0.15 ± 0.52 D in the treated DED group and 0.14 ± 0.51 D in the control group. Median absolute error (MedAE) was similarly low (0.034 D vs. 0.023 D), and mean bias error (MBE) remained close to zero in both groups (0.003 D vs. 0.002 D). There was no statistically significant difference in absolute prediction error between treated DED eyes and controls (Mann–Whitney U = 1616; *p* = 0.847).

### 3.6. Ocular Surface Parameters Before and After Surgery in the DED Group

**Figure 9:**
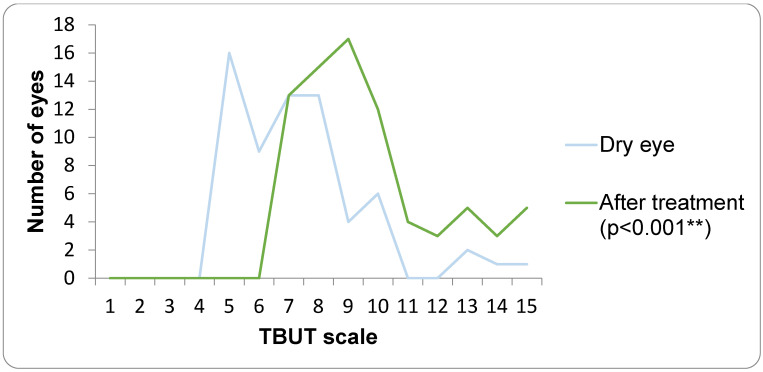
TBUT before and after treatment. ** statistically significant difference.

**Figure 10:**
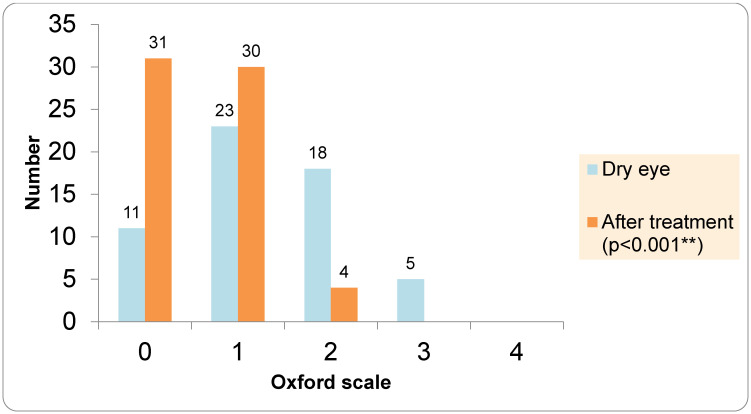
Oxford scale before and after treatment. ** statistically significant difference.

In the DED group, dry eye parameters showed significant improvement following four weeks of treatment with lipid-containing artificial tears. TBUT increased significantly after treatment (Wilcoxon signed-rank test, *p* < 0.001; Figure 9), indicating improved tear film stability. Similarly, Meibum Quality Score and Oxford scale demonstrated a significant shift toward lower severity grades after treatment (*p* < 0.001; Figure 10).

## 4. Discussion

This cross-sectional study evaluated the impact of dry eye disease (DED) on the stability of keratometric measurements used for intraocular lens (IOL) power calculation in cataract patients and assessed whether a four-week course of lipid-containing artificial tears could improve keratometric reproducibility and refractive prediction. Consistent with the high prevalence of ocular surface disease in the cataract population, more than half of our cohort fulfilled diagnostic criteria for DED, most commonly with features of MGD–related evaporative disease, in line with previous reports documenting MGD in approximately half of patients presenting for cataract surgery [17,18].

In our study, eyes with DED exhibited greater variability of keratometric astigmatism between repeated measurements compared with controls, whereas mean keratometric power and astigmatic axis remained relatively stable. This pattern is in agreement with prior work demonstrating that an unstable tear film preferentially degrades the regularity of the corneal surface and thus the magnitude and orientation of anterior corneal astigmatism. Epitropoulos et al. showed that patients with hyperosmolar tears had significantly greater inter-visit variability in average keratometry and vector magnitude of anterior corneal astigmatism, which translated into clinically relevant differences in calculated IOL power [10]. Similarly, Hiraoka et al. reported reduced repeatability of corneal curvature radius in cataract patients with DED, while axial length measurements remained largely unaffected, underscoring the particular vulnerability of anterior corneal measurements to tear film instability [17]. Our findings extend these observations by demonstrating that astigmatic instability persists even when modern optical biometry and a single IOL platform are used, and by quantifying its downstream influence on IOL power calculation sets (IOL 1, IOL 2, IOL 3).

The clinical relevance of these keratometric fluctuations lies in their potential to generate postoperative refractive surprises. Systematic evidence indicates that keratometric error accounts for a substantial proportion of residual refractive error after cataract surgery, and that ocular surface disease, particularly DED, is one of the major upstream causes. In the systematic review by Biela et al., all included studies that treated DED before surgery demonstrated a reduction in mean absolute error (MAE) compared with biometry obtained prior to ocular surface optimization [18]. Our data are in line with this concept: within the DED group, the third IOL calculation (IOL 3), obtained after four weeks of targeted tear film therapy, differed modestly but significantly from pre-treatment calculations, indicating that the “true” keratometric profile used for surgery was only revealed after ocular surface rehabilitation.

Short-term effects of artificial tears on corneal measurements have been a source of concern, particularly when biometry is performed immediately after drop instillation. Röggla et al. found that both low- and high-viscosity artificial tears induced a significant increase in short-term variability of keratometric readings in cataract patients, with changes persisting up to five minutes and being more pronounced in eyes with DED [6]. Other studies using aberrometry and straylight metrics have confirmed that various dry-eye drops temporarily worsen optical quality and increase higher-order aberrations in the minutes following instillation, especially with more viscous formulations [11,19]. Together, these data suggest that while artificial tears can improve the ocular surface in the longer term, they may transiently introduce noise into keratometry and wavefront measurements if used immediately before testing. In our protocol, biometry used for surgical planning was performed at least several hours after the last instillation of artificial tears, and “IOL 3” represents the effect of chronic tear film optimization rather than an acute pharmacologic perturbation, which likely explains why we observed a shift in mean IOL power but not an increase in measurement variability at the final visit.

A particular feature of our work is the use of a lipid-containing artificial tear in a cohort with a substantial proportion of Evaporative dry eye (EDE). Current pathophysiologic models identify MGD as the leading cause of EDE, with reduced or altered meibum disrupting the tear film lipid layer and accelerating evaporation, tear film breakup, and ocular surface inflammation [19,20]. Lipid-based eye drops are designed to supplement or mimic this deficient lipid layer, thereby reducing evaporation and stabilizing the tear film. A recent review by Maulvi et al. summarized evidence that mineral oil, castor oil, phospholipid-, and triglyceride-containing formulations can enhance lipid layer thickness, prolong tear breakup time (TBUT), and improve both signs and symptoms in patients with EDE, particularly those with MGD [15]. Our study adds to this body of work by linking a four-week course of a lipid-containing artificial tear not only to improvements in classical DED parameters (OSDI, TBUT, staining, Meibomian gland function) but also to measurable changes in the keratometric data that underpin IOL power calculation.

Despite the demonstrated influence of DED and its treatment on keratometry and predicted IOL power, the final refractive outcomes in our cohort were favorable in both DED and control groups, with comparable absolute prediction errors. This apparent discrepancy likely reflects several factors. First, the absolute differences between pre- and post-treatment IOL calculations in the DED group were small (on the order of 0.1 D on average), and thus below the threshold that would translate into large clinical refractive shifts in most eyes, especially when non-toric monofocal lenses are used. Second, modern formulas such as Barrett Universal II exhibit a degree of robustness to small keratometric perturbations, particularly in eyes without extreme axial lengths. Third, our protocol incorporated repeated keratometric measurements and mandated ocular surface optimization before final IOL selection in the DED group, which may have mitigated the potential impact of baseline tear film instability on postoperative refraction. These observations are broadly consistent with the literature synthesized by Biela et al., where DED treatment consistently reduced—but did not always eliminate—residual error, and with studies in which targeted therapies (e.g., cyclosporine, lifitegrast, diquafosol, or loteprednol) improved the accuracy of refractive prediction without fundamentally changing the distribution of excellent outcomes [18].

Our findings complement emerging interventional data suggesting that more aggressive preoperative treatment of ocular surface disease can further enhance refractive accuracy. Kawagoe et al. recently reported that intense pulsed light with Meibomian gland expression (IPL-MGX) in patients with MGD-related DED significantly improved the accuracy of predicted spherical equivalent after cataract surgery, attributing this to better tear film stability and more reliable keratometry [21]. Other groups have documented similar benefits from pharmacologic DED therapies on preoperative biometry and refractive outcomes [18]. Against this background, our data support a “stepwise” approach: for mild to moderate DED, regular use of lipid-containing artificial tears and lid hygiene may suffice to stabilize measurements, while more advanced or refractory cases might require office-based MGD treatments or anti-inflammatory agents to achieve similar reliability.

From a practical standpoint, several clinical implications arise. First, systematic screening for DED and MGD in cataract candidates using standardized questionnaires and objective tests (e.g., TBUT, staining, Schirmer, lid margin and meibum assessment) should be integrated into routine preoperative work-up, as recommended by contemporary algorithms for ocular surface management [17,18]. Second, biometry should ideally be performed on a stable tear film: measurements taken immediately after instillation of artificial tears-particularly viscous or lipid-rich formulations-are best avoided given the evidence of transient disturbances in optical quality and keratometry [6,11]. Third, in patients with documented DED, especially evaporative disease, a period of preoperative tear film optimization (such as the four-week course used in our study) appears justified to reduce astigmatic variability and refine IOL power selection, even if final refractive outcomes are expected to be good with modern technologies.

This study has limitations. It was conducted at a single center with a moderate sample size, which may limit generalizability. The severity of DED was predominantly mild to moderate; our findings may not extrapolate to severe ocular surface disease or patients with significant cicatricial pathology. In addition, the relatively small number of patients within individual DED subtypes, particularly aqueous-deficient dry eye, precluded adequately powered subgroup analyses comparing treatment effects across DED phenotypes. Only one type of monofocal IOL and a single modern formula (Barrett Universal II) were used, so the magnitude of DED-related effects might differ for toric or multifocal lenses, in which small keratometric deviations can be more clinically significant.

In addition, refractive accuracy was primarily evaluated using mean and median absolute prediction error, while other metrics such as the root mean square absolute error were not included. Although RMSAE has been proposed as a complementary measure [22], particularly in datasets with greater dispersion or larger outliers, the relatively small prediction errors observed in our cohort support the use of MAE and MedAE as clinically interpretable outcome measures. Furthermore, the DED group received a specific lipid-containing artificial tear regimen without randomization to alternative therapies or placebo, precluding a direct comparison of different treatment strategies. The follow-up period was limited to one month postoperatively; longer-term outcomes, including stability of refraction and ocular surface status, were not assessed. Finally, the study did not assess the potential postoperative refractive error that might have resulted from selecting intraocular lens power based on the first or second preoperative keratometric measurement in patients with dry eye disease, which would require a prospective design with systematic comparison of postoperative outcomes.

## 5. Conclusions

This cross-sectional study highlights how even modest disturbances of the tear film in dry eye disease can subtly—but meaningfully—undermine the reproducibility of keratometric astigmatism in patients preparing for cataract surgery. While spherical keratometric values remained largely unaffected, variability in astigmatism was sufficient to shift predicted intraocular lens power, underscoring the sensitivity of modern biometry to ocular surface instability.

A structured, four-week regimen of lipid-containing artificial tears produced measurable improvements in tear-film stability, ocular surface health, and the consistency of keratometric readings. Once the ocular surface was rehabilitated, IOL calculations became more stable, yet final postoperative refractive accuracy remained high and comparable to that of controls—suggesting that optimization rather than correction is the key benefit of preoperative treatment.

Taken together, these findings reinforce the value of systematically identifying and managing dry eye disease before cataract surgery. Ensuring a stable tear film, performing measurements away from the transient effects of drop instillation, and confirming keratometric consistency through repeated biometry appear to be practical steps that can refine preoperative planning and support reliable refractive outcomes. Integrating these measures into routine cataract pathways may help reduce the risk of avoidable variability in IOL power selection and enhance the overall precision of contemporary cataract surgery.

## Figures and Tables

**Table 1:** Study population: DED vs. Control group.

Parameter	DED g65	Control 51
Sex (F/M)	43/22	26/25
Age (years), mean ± SD	76.46 ± 7.55	74.29 ± 6.87

**Table 2:** Dry eye classification within the DED group (*n* = 65).

DED Characteristic	Value
Aqueous-deficient DED (ADDE)	18%
Evaporative dry eye (EDE)	37%
Mixed-type DED	45%
OSDI categories	
—Normal	21.5%
—Mild	37.0%
—Moderate	41.5%
—Severe	0% (not observed)

## Data Availability

The data supporting the findings of this study are not publicly available due to the presence of personal information and ethical considerations.

## Abbreviations

The following abbreviations are used in this manuscript:

DED	Dry Eye Disease
IOL	Intraocular Lens
DEWS	Dry Eye Workshop
OSDI	Ocular Surface Disease Index
TBUT	Tear Breakup Time
MGD	Meibomian Gland Dysfunction
K	Keratometry
Avg K	Average Keratometry
Ast K	Corneal Astigmathism
HLB	Hydro Lipid Balance
JGL	Jadran Galenski Laboratorij
PE	Prediction Error
MAE	Mean Absolute Error
MedAE	Median Absolute Error
MBE	Mean Bias Error
ADDE	Aqueous Deficient Dry Eye
EDE	Evaporative Dry Eye
MQS	Meibum Quality Score
